# Gut integrity in critical illness

**DOI:** 10.1186/s40560-019-0372-6

**Published:** 2019-03-20

**Authors:** Shunsuke Otani, Craig M. Coopersmith

**Affiliations:** 1 Department of Surgery and Emory Critical Care Center, Emory University School of Medicine, 101 Woodruff Circle, Suite WMB 5105, Atlanta, GA 30322 USA; 2 Department of Emergency and Critical Care Medicine, Chiba University Graduate School of Medicine, Chiba, Japan; 3 Department of General Medical Science, Graduate School of Medicine, Chiba University, 1-8-1 Inohana, Chuo-ku, Chiba City, Chiba 260-8670 Japan

**Keywords:** Gut, Intestine, Bacteria, Sepsis, Intensive care unit, Microbiome, Critical illness, Epithelium, Pathobiome, Apoptosis

## Abstract

**Background:**

The gut is hypothesized to be the “motor” of critical illness. Under basal conditions, the gut plays a crucial role in the maintenance of health. However, in critical illness, all elements of the gut are injured, potentially worsening multiple organ dysfunction syndrome.

**Main body:**

Under basal conditions, the intestinal epithelium absorbs nutrients and plays a critical role as the first-line protection against pathogenic microbes and as the central coordinator of mucosal immunity. In contrast, each element of the gut is impacted in critical illness. In the epithelium, apoptosis increases, proliferation decreases, and migration slows. In addition, gut barrier function is worsened via alterations to the tight junction, resulting in intestinal hyperpermeability. This is associated with damage to the mucus that separates the contents of the intestinal lumen from the epithelium. Finally, the microbiome of the intestine is converted into a pathobiome, with an increase in disease-promoting bacteria and induction of virulence factors in commensal bacteria. Toxic factors can then leave the intestine via both portal blood flow and mesenteric lymph to cause distant organ damage.

**Conclusion:**

The gut plays a complex role in both health and critical illness. Here, we review gut integrity in both health and illness and highlight potential strategies for targeting the intestine for therapeutic gain in the intensive care unit.

## Background

The gut has long been hypothesized to be the “motor” of critical illness [1–6]. The original biological explanation behind this theory is that critical illness induces intestinal hyperpermeability, leading to bacterial translocation via the portal circulation, leading to subsequent systemic infection and distant organ damage. In addition, studies over the past 20 years have demonstrated multiple additional methods in which the intestine can drive both local and distant injury.

In isolation, each component of the gut is severely compromised by critical illness, leading to both local and distant organ damage. Further, crosstalk between components of the gut and distant organs exacerbates cellular and organ injury. This review describes gut integrity in health and in critical illness, and potential ways in which the gut integrity can potentially be targeted as a therapeutic target in the ICU.

## The gut in health

### The intestinal epithelium

The gut is covered by a single-cell layer epithelium with a surface area of 30 m^2^, similar in size to half a badminton court [7]. The intestinal epithelium plays a role in managing host homeostasis and plays a critical role as the first-line protection against pathogenic microorganisms and as the central coordinator of mucosal immunity [8]. The intestinal epithelium also communicates with gut-associated lymph tissue and produces hormones, cytokines, and antimicrobial peptides. The single-cell layer intestinal epithelium is covered by a mucus layer which prevents direct contact between the epithelium and luminal contents [9].

The gut is a continuously renewing organ with the majority of cells turning over within 1 week. Intestinal stem cells reside near the base of crypts of Lieberkühn and can be identified by staining for the biomarker leucine-rich repeat-containing G protein-coupled receptor (Lgr) 5, which is present in stem cells but not the surrounding Paneth cells. Renewal and differentiation of Lgr5^+^ intestinal stem cells is modulated by clusters of differentiation (CD) 4^+^ T helper cells [10], whereas their apoptosis and regeneration are mediated by the protein ARTS (a Septin-4 isoform) which induces cell death by translocating from the mitochondria to the cytosol where it binds to and antagonizes the X-linked inhibitor of apoptosis [11]. In addition, intestinal stem cells express the transmembrane protein toll-like receptor (TLR) 4, which regulates their proliferation and apoptosis through activation of p53-upregulated modulator of apoptosis (PUMA), which interacts with anti-apoptotic factors, thereby allowing pro-apoptotic signaling to the mitochondria [12].

Intestinal stem cells migrate up the crypt where they divide into daughter cells. Upon leaving the crypt, small intestinal epithelial cells enter the villus where they differentiate into (a) absorptive enterocytes (by far the most numerous cell type); (b) mucus-producing goblet cells, which also secrete anti-microbial proteins, chemokines, and cytokines [13]; (c) hormone-producing enteroendocrine cells; and (d) tuft cells which initiate mucosal immunity against parasitic infections [14]. As cells reach the tip of the villus, they either die by apoptosis or are exfoliated whole into the lumen. In contrast to other intestinal epithelial cell types, Paneth cells migrate downwards to the base of the crypts. Paneth cells secrete several antimicrobial peptides such as defensin [15] and are longer lived than other gut epithelial cells.

### The intestinal immune system

The intestinal immune system is a remarkably complex and diverse ecosystem and has more lymphocytes than any other location in the body. Recent years have brought numerous new insights into crosstalk between the mucosal immune system and both the intestinal epithelium and the microbiota [16]. The intestine contains the largest population of lymphocytes in the body. CD4^+^ T cells modulate intestinal epithelial function and enhance production of antimicrobial peptides during infection, leading to pathogen eradication [17]. Intraepithelial lymphocytes are antigen-experienced T cells located within the gut epithelium that have immediate access to antigen in the gut lumen [18]. Secretive IgA, which recognizes and coats commensal bacteria, is derived from plasma cells of the germinal center and is abundant in the lamina propria, representing 80% of all immunoglobulin produced in the body. In addition, innate lymphoid cells, mucosa-associated invariant T cells, and cells of the mononuclear phagocyte systems all play a role in mucosal immunity [16].

### Intestinal microbiota

Approximately 40 trillion microorganisms reside within the intestine [19], and the number of genes within the microbiome is exponentially larger than that of the human genome [20]. The gut microbiome contains approximately 1000 different species, weighs 1.5 kg, and contains more DNA than every host organ combined [21]. Recent advances in metagenomic sequencing and mass spectrometry reveal that the microbiome plays a pivotal role in maintaining health and homeostasis [22].

## The gut in critical illness

### Epithelial alterations and intestinal hyperpermeability

Apoptosis is upregulated in intestinal epithelial cells following both cecal ligation and puncture and *Pseudomonas pneumonia* in mice [23, 24]. Notably, prevention of gut apoptosis by overexpression of B-cell lymphoma 2 (Bcl-2) improves survival in both of these models. In contrast, sepsis induces a profound decrease in crypt proliferation [25]. Migration up the crypt/villus axis is also slowed by critical illness resulting in a marked diminution of villus length [26, 27]. The molecular determinants underlying this are complex with migration occurring more rapidly in mice lacking TLR4 in necrotizing enterocolitis but more slowly in septic mice lacking TLR4. In addition, blocking phosphorylated focal adhesion kinase (P-FAK) leads to a further slowing of enterocyte migration, whereas overexpression of gut-specific Bcl-2 prevents sepsis-induced slowing of enterocyte migration.

Critical illness also induces hyperpermeability of the gut barrier which begins as early as 1 h after the onset of sepsis and lasts at least 48 h [28–32]. This impaired barrier function is mediated by changes in the tight junction and associated proteins and allows outflow of luminal contents and likely damages distant organs. Mechanistically, claudin-2 and junctional adhesion molecule (JAM)-A are increased by sepsis, whereas claudin-5 and occludin are decreased by sepsis. Zonula occludens (ZO)-1 is also variably decreased depending on model system [30, 32–34]. In addition, myosin light chain kinase (MLCK) phosphorylates the myosin regulatory light chain, resulting in contraction of the actin-myosin ring, increasing paracellular permeability. MLCK activation is commonly found with bacterial infection [35, 36], and inhibition of MLCK improves survival in a mouse model of sepsis [37] as well as improving barrier function and tight junction rearrangement in a murine model of burn injury [38]. Of note, changes to the gut epithelium and barrier function are exacerbated in the presence of chronic co-morbidities such as cancer [39, 40] or chronic alcohol use [41–43].

Mucus also plays a crucial role in host defense by preventing bacteria and digestive enzymes from coming into contact with the gut epithelium, and the hydrophobic properties of mucus significantly decrease the ability of positively charged, water-soluble toxic molecules to traverse the surface [44]. The mucus layer is damaged during critical illness, which, in turn, results in epithelial cell dysfunction. Ischemia/reperfusion leads to a loss of hydrophobicity of the mucus layer and altered intestinal permeability [44]. In addition, after trauma/hemorrhagic shock, rats have decreased mucus and villus height loss with increased epithelial apoptosis and hyperpermeability [28]. Notably, H2 blockers decrease gut mucus production and lead to barrier dysfunction in vitro [45].

### The pathobiome

The density and composition of the microbiota are drastically altered within hours of the onset of critical illness with the conversion of the health-inducing microbiome into a disease-promoting pathobiome [46]. Significant emerging data suggests a link between critical illness and the microbiome. The largest study in the field of critical care examined microbiota in the skin, tongue, and stool of 115 intensive care unit (ICU) patients within 48 h of ICU admission and ICU discharge or 10th ICU day to over 1000 patients from the American Gut Project [47]. Alpha-diversity (within group) of stool and skin was considerably decreased at ICU admission. At the phylum level, the relative abundance of *Firmicutes* and *Bacteroidetes* was decreased, whereas *Proteobacteria* was increased in the stool of ICU patients. At the genus level, *Faecalivacterium*, which has anti-inflammatory properties, was massively decreased, but common pathogens *Enterobacter* and *Staphylococcus* were increased. Complementary results were demonstrated in a prospective study of 34 ICU patients that showed a significant decrease in *Firmicutes* and *Bacteroidetes* and an increase in *Proteobacteria* compared to 15 healthy controls at the phyla level [48]. At the genus level, *Faecalibacterium*, *Blautia*, *Ruminococcus*, *Subdoligranulum*, and *Pseudobutyrivibrio* were all significantly decreased, and overall microbiota diversity was significantly impaired. A loss of microbiota diversity was also observed in a smaller study of 14 septic ICU patients where remarkably 35% of patients had only 1 to 4 bacterial taxa in their stool [49]. Overall, *Proteobacteria* was dominant in the ICU, and the number of *Firmicutes* decreased, whereas *Enterococcus*, *Staphylococcus*, and *Enterobacter* all increased in septic patients. Of note, under basal conditions, the taxa within the gut microbiome are relatively temporally stable although may be impacted by diet and environmental factors [50]. In contrast, the transition to a pathobiome occurs nearly immediately in ICU patients [51]. Dysbiosis progression has also been observed in pediatric ICU patients [52].

The etiology of the instability in the microbiome in critical illness is multifactorial. Critical illness, in isolation, causes profound alterations to the gut microbiota, likely caused by the overall alterations in host milieu. Notably, bacteria can become newly virulent in the setting of critical illness by expressing ancestral or newly acquired genes [53, 54]. In addition, numerous treatments delivered to patients in the ICU have off-target effects that directly alter the microbiome. Drugs that have been shown to impact the microbiome include antibiotics, proton pump inhibitors, and opioids [55, 56]. In addition, nutrition components (carbohydrates, lipid, and protein) and route (enteral/parenteral) alter the microbiome in health [57–59]. Little information is available as to the role nutrition plays on the microbiome in critical illness [60], although a murine study demonstrated increased *Bacteroidetes* and impaired barrier function following parenteral nutrition, which was reversed by enteral nutrition supplementation [61].

### Gut lymph hypothesis

The gut lymph hypothesis states that noxious mediators originating from the intestinal lumen travel via the mesenteric lymph to the lung where they cause tissue damage. Several pieces of research support this hypothesis. Ligating the mesenteric lymph duct decreases lung injury and attenuates neutrophil activation in rodent models of critical illness with improved survival [62, 63]. Further, injecting mesenteric lymph from trauma-hemorrhage induces lung hyperpermeability and lung injury [64]. Complementary to this is the gut-lung axis of critical illness [65]. Lung communities are dominated by gut-associated bacteria following murine sepsis, and ecological analysis revealed the gut as the likely source of lung bacteria. This is consistent with the abundance of gut-specific bacteria in ICU patients with acute respiratory distress syndrome [66].

## Therapeutic approaches targeting the intestine

### Gut epithelial integrity and permeability—basic research

No current therapy exists to target the gut epithelium, permeability, or mucus at the bedside of critically ill patients. However, multiple pre-clinical strategies exist that may be potential targets in the future. For example, epidermal growth factor (EGF) has been shown to improve gut apoptosis, proliferation, and permeability following either cecal ligation and puncture or *Pseudomonas pneumonia* even if started 24 h after the onset of sepsis [42, 67–69]. Additionally, a membrane permeant inhibitor of MLCK improves intestinal permeability and prevents occludin and ZO-1 reduction following acute alcohol intoxication and burn injury in mice [70]. Administration of a mucus surrogate also prevents trauma/hemorrhagic shock-induced gut injury [71]. In addition, treatment with a pharmacologic vagus nerve agonist attenuates lung injury caused by toxic mesenteric lymph following trauma/hemorrhagic shock in rats [72].

### Microbiome—clinical research

Conceptually, the microbiome can be targeted by increasing “health-promoting” bacteria, decreasing “disease-promoting” bacteria or preventing an alteration in bacterial virulence factors. Many of these have been tried in patients with variable results.

Probiotics are exogenous live bacteria administered to the host and have been extensively studied in critical illness. Meta-analyses have demonstrated a decrease in ventilator-associated pneumonia following administration of probiotics, but this has not been accompanied by alterations in mortality or length of stay [73–75]. The evidence is not high quality, however, as the studies have been limited by significant heterogeneity in terms of types of bacteria used, timing of administration, and quality of the studies as well as the fact that the majority of these studies were performed prior to more recent breakthroughs in microbiome analysis. Notably, 4-week administration of probiotics to healthy volunteers does not alter gut microbiota [76]. However, there are questions about sustainability after probiotic administration as well as geographic variation in the intestine whereby the microbiota might resist or enhance colonization based upon probiotic effects [77]. Furthermore, when humans (and mice) are given antibiotics and then given autologous microbiome transplantation (pre-antibiotic stool) or probiotics, one’s own stool rapidly reconstituted a normal microbiome but probiotics were associated with a marked delay in return to normal microbiome [78].

Unlike the selective approach of probiotics, fecal microbial transplantation (FMT) is a strategy in which the entire microbiome is transplanted from a healthy donor, with the goal of reconstructing normal commensal flora in the diseased gut. FMT has been demonstrated to be remarkably successful in the treatment of recurrent *Clostridium difficile* infection with a 92% response rate to treatment [79]. FMT is also increasingly being used for dysbiosis caused by other intestinal pathology (such as inflammatory bowel disease). The intermediate long-term effects of FMT on the microbiome are not clear as studies to date have shown conflicting results [80, 81]. To date, data on FMT in the ICU is limited to case reports [82], and its safety and efficacy are currently unknown. Further, many ICU patients receive antimicrobial therapy, which would be expected to alter the microbiome after FMT is performed. As such, FMT should be considered experimental in critical illness currently.

Selective decontamination of the digestive tract (SDD) takes the opposite to probiotics and FMT by targeting pathogenic gut bacteria. SDD has been shown to be effective at improving mortality in multiple studies and meta-analyses originating from environments with low anti-microbial resistance [83]. SDD continues to be controversial because of theoretical concerns that it could induce multidrug resistance [84]. Importantly a recent study randomized over 8000 patients on mechanical ventilation in 13 ICUs with moderate to high levels of antibiotic resistance to (a) a modified version of SDD (without a 4-day course of intravenous antibiotics), (b) selective oropharyngeal decontamination, and (c) chlorhexidine mouthwash and compared them to a baseline period [85]. No reduction in ICU-acquired bloodstream infection or mortality was detected in any of the groups compared to baseline.

### Microbiome—basic research

No bedside therapy exists to prevent induction of new virulence factors in bacteria. However, bench research suggests that bacterial sense intraluminal phosphate, and a lack of phosphate, plays a critical role in the induction of virulence [86]. As such, repletion of intraluminal (not intravenous) phosphate has the potential to trick bacteria into “believing” that a diseased host is healthy. Preclinical data demonstrates that enterally administered polyethylene glycol-conjugated phosphate improves survival in murine intraabdominal sepsis [86].

## Conclusions

**Fig. 1:**
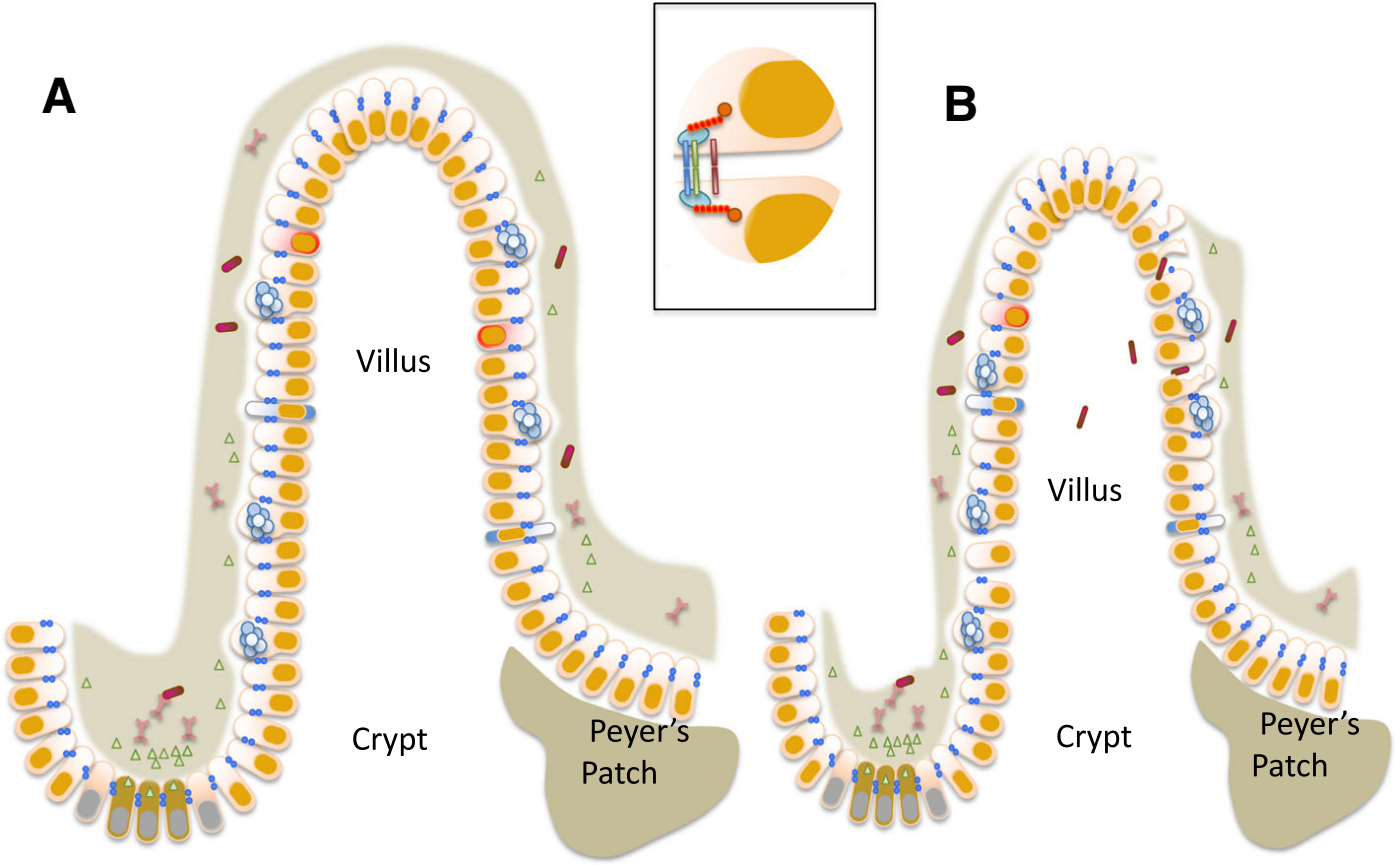
The gut in health and critical illness. In conditions of health (**a**), intestinal stem cells proliferate in the crypt (gray and orange), divide into daughter cells, and migrate up in a single-cell layer to the top of the villus. The majority of epithelial cells are enterocytes (white and orange), although there are also goblet cells, enteroendocrine cells, and tuft cells present. The epithelium is surrounded by a continuous mucus layer (gray). This acts as a barrier to luminal microbes (red and green) which are also recognized by secretive IgA (light red). Permeability is also mediated via the tight junction (inset) where a complex machinery between epithelial cells acts as a selective barrier allowing solutes and water through but preventing movement of larger molecules. In critical illness (**b**), proliferation is decreased and apoptosis is increased leading to a shorter villus length. The mucus layer is damaged and no longer uniform. Along with changes in the tight junction resulting in hyperpermeability, gut barrier function is compromised and bacteria are able to translocate (red rods representing bacteria are present in the lamina propria)

All elements of the gut—the epithelium, mucus, the immune system, and the microbiome—are profoundly altered by critical illness compared to health (Fig. 1). Insults to the gut can, in turn, lead to local and distant injury and multiple organ dysfunction syndrome. While therapeutic approaches targeting most of these are several years away from the bedside, several therapeutic approaches currently exist to target the pathobiome. However, none of these is currently standard of care in the ICU, and further research is needed to determine how to target intestinal injury in critical illness.

## Abbreviations

Bcl-2: B-cell lymphoma 2
CD4: Clusters of differentiation 4
EGF: Epidermal growth factor
FMT: Fecal microbial transplantation
ICU: Intensive care unit
JAM-A: Junctional adhesion molecule A
Lgr5: Leucine-rich repeat-containing G protein-coupled receptor 5
MLCK: Myosin light chain kinase
P-FAK: Phosphorylated focal adhesion kinase
PUMA: P53-upregulated modulator of apoptosis
SDD: Selective decontamination of the digestive tract
TLR: Toll-like receptor
ZO-1: Zonula occludens-1
